# Nutritional Support via Jejunostomy Placed During Staging Laparoscopy for Esophagogastric Cancer: A Case Series

**DOI:** 10.3390/healthcare14010089

**Published:** 2025-12-30

**Authors:** Maria Tieri, Claudia Sivieri, Jacopo Viganò, Salvatore Corallo, Andrea Dagnoni, Anna Pagani, Elisa Mattavelli, Anna Uggè, Francesca De Simeis, Alice Tartara, Paolo Pedrazzoli, Riccardo Caccialanza, Valentina Da Prat

**Affiliations:** 1 Department of Experimental Oncology, IEO, European Institute of Oncology IRCCS, 20141 Milan, Italy; maria.tieri@ieo.it; 2 Clinical Nutrition and Dietetics Unit, Fondazione IRCCS Policlinico San Matteo, 27100 Pavia, Italy; claudia.sivieri01@universitadipavia.it (C.S.); e.mattavelli@smatteo.pv.it (E.M.); a.ugge@smatteo.pv.it (A.U.); f.desimeis@smatteo.pv.it (F.D.S.); a.tartara@smatteo.pv.it (A.T.); r.caccialanza@smatteo.pv.it (R.C.); v.daprat@smatteo.pv.it (V.D.P.); 3 General Surgery 1, Fondazione IRCCS Policlinico San Matteo, 27100 Pavia, Italy; j.vigano@smatteo.pv.it; 4 Department of Oncology, Comprehensive Cancer Center, Fondazione IRCCS Policlinico San Matteo, 27100 Pavia, Italy; a.pagani@smatteo.pv.it (A.P.); p.pedrazzoli@smatteo.pv.it (P.P.); 5 Department of Clinical, Surgical, Diagnostic & Pediatric Sciences, University of Pavia, 27100 Pavia, Italy; 6 General Surgery Residency Program, University of Pavia, 27100 Pavia, Italy; andrea.dagnoni01@universitadipavia.it; 7 Department of Oncology and Hematology-Oncology, University of Milan, 20122 Milan, Italy

**Keywords:** esophagogastric cancers, feeding jejunostomy, staging laparoscopy, nutritional assessment, nutritional support, home enteral nutrition, weight loss

## Abstract

**Background**: Malnutrition is associated with poorer clinical outcomes in esophagogastric cancers (EGCs). Enteral nutrition via feeding jejunostomy (FJ) is feasible and effective, although standardized criteria for its placement during staging laparoscopy (SL) are lacking. Here, we describe a case series with the aim of generate preliminary evidence in highlighting unmet needs in this setting. **Methods**: We retrospectively reviewed medical records of EGC patients who underwent FJ placement during SL at the Fondazione IRCCS Policlinico S. Matteo from January 2022 to December 2023. Patients with missing nutritional data or known metastatic disease were excluded. **Results**: We included 14 Caucasian patients aged 66 years (IQR: 56.3–69.5) with a median Body Mass Index (BMI) of 23.7 kg/m^2^ (IQR: 21.6–26.3). The tumor location was the gastroesophageal junction in eight cases (57%), the body of the stomach in four cases (29%), and the esophagus in two cases (14%). At the time of diagnosis, all patients had experienced weight loss: 13.4% of body weight (IQR: 8.7–16.8) in the last 6 months; with high malnutrition risk scores: NRS-2002 = 3 (IQR: 2–4) and MUST = 2 (IQR: 1–2). Prior to FJ placement only four (29%) patients had tried oral nutrition supplements (ONS) and nine (64%) had been evaluated by dietitians. Home enteral nutrition (HEN) was started in twelve (86%) cases, with three (21%) providing total enteral nutrition and 9 (64%) as supplemental HEN, providing a median of 45.5% of energy needs (IQR: 32.6–68.2). Due to sufficient oral intake, HEN was not started in two cases (14%) and was discontinued in the first month in another two cases. In this series, FJ was in place but unused for a median duration of 11 days (IQR: 3–91). The median duration of HEN was 97 days (IQR: 40–135); with 5 (35%) patients achieving weight stability/gain. FJ-related complications requiring hospitalization occurred in three (21%) cases. **Conclusions**: In this case series, we observed a suboptimal utilization of the FJ. Several patients had not undergone ONS trials or dietitian assessment prior to FJ placement, while others retained the FJ for months without using it. Given the potential risks of FJ, standardized selection criteria are warranted; routine preoperative nutritional assessments before SL should be implemented to identify high-risk patients and optimize FJ placement.

## 1. Introduction

Esophagogastric cancers (EGCs), which include cancers of the esophagus, esophagogastric junction, and stomach, represent a significant global health concern. According to GLOBOCAN data, esophageal cancer (EC) is the 11th most common diagnosed cancer worldwide, ranking 7th in terms of mortality overall with 445,129 deaths. Gastric cancer (GC) represents the 5th most common cancer globally, being responsible for an estimated 659,853 deaths, ranking 5th for mortality globally [1].

Disease stage of EGCs determines the therapeutic approach, which has progressively become patient and disease-tailored [2,3,4]. Surgery is the primary treatment for the majority of patients with resectable EGC. However, despite the improvement in surgical procedures, most patients experience disease recurrence after resection [5]. Multiple therapeutic strategies, such as perioperative chemotherapy and adjuvant or neoadjuvant chemoradiation, have been developed to enhance treatment outcomes for patients with resectable disease. These approaches have demonstrated improved survival compared to surgery alone [6,7,8,9]. Notably, the FLOT4 trial showed that eight cycles of perioperative 5-fluorouracil, leucovorin, oxaliplatin, and docetaxel (FLOT) improved both overall and disease-free survival compared with epirubicin and cisplatin plus either fluorouracil or capecitabine in patients with locally advanced resectable EGCs [10]. Based on these results, this regimen has become the standard of care for resectable patients who can tolerate triple cytotoxic therapy, particularly in Western countries [5]. More recently, the phase 3 multinational MATTERHORN trial found that adding the anti-programmed death ligand 1 (PD-L1) agent durvalumab to FLOT significantly improved event-free survival compared to FLOT alone in patients with resectable EGCs [11]. This combination may become the new global standard of care in the near future.

Disease-related malnutrition significantly affects cancer treatment and outcomes, leading to treatment delays, higher toxicity, more surgical complications, poorer prognosis, and reduced quality of life [12]. For instance, in gastric cancer patients, poor nutritional status, reflected by high controlling nutritional status (CONUT) or low prognostic nutritional index (PNI) scores, has been associated with lower survival rates and increased postoperative complications [13,14]. EGCs pose significant nutritional challenges due to tumor-related symptoms and treatment side effects, leading to substantial weight loss and reduced physical performance. In EC, dysphagia, systemic inflammation, and therapies contribute to cachexia and malnutrition [15,16,17], while in GC, reduced intake due to stenosis, metabolic alterations, and post-gastrectomy syndromes like dumping syndrome and pancreatic insufficiency worsen malnutrition [18,19,20]. Although overweight may be associated with higher rates of anastomotic leakage after esophagectomy, no clear survival worsening has been observed in overweight EC patients [21,22]. According to recent evidence, obesity may paradoxically be linked to improved survival in gastrointestinal cancer [23].

Recently, the Global Leadership Initiative on Malnutrition (GLIM) has established a global consensus on the diagnosis of malnutrition, combining phenotypic criteria (unintentional weight loss, low Body Mass Index (BMI), or reduced muscle mass) with etiologic criteria (reduced food intake or inflammation/disease burden) [24]. However, the application of these rigorous criteria in clinical practice, particularly regarding muscle mass assessment, remains challenging.

Given the widespread occurrence and significant consequences of malnutrition among EGC patients, there is a critical need to prioritize early detection and effective nutritional interventions [25,26]. Despite the importance of appropriate nutritional support as an integral part of EGC care [26,27], no standardization exists with regard to timing, route, and dosage.

There are several approaches to provide nutritional support for EGCs, and the specific clinical context determines the routes, timing, and aims of the interventions. The choice is closely related to the patient’s nutritional status, gastrointestinal integrity and accessibility, type of treatment being administered, and related side effects, as well as the overall prognosis [28].

Enteral nutrition is recommended whenever per os intakes are not possible or not sufficient in patients with EGCs. Among the enteral accesses, feeding jejunostomy (FJ) provides a stable route for delivering nutrients directly into the small intestine. FJ is typically placed during staging laparoscopy (SL) or curative surgery, especially in cases of obstruction, malnutrition, or expected prolonged fasting [29]. While some studies report FJ is both safe and effective in maintaining nutritional status and favoring treatment completion [30,31,32,33,34], others find no significant improvement in clinical outcomes, questioning its routine use [35,36,37,38,39]. In GC, although some studies highlight increased postoperative complications risk without improved chemotherapy adherence [40,41], many others underline benefits such as reduced morbidity, shorter hospital stays, and improved nutritional status and recovery [42,43,44,45,46].

Collectively, existing studies provide inconclusive evidence on the benefits of routine FJ for nutritional and clinical outcomes in EGCs, with limited data on patients’ baseline nutritional status, timing, and duration of support [47,48]. Utilization of FJ is not without risks or complications [49], and current evidence lacks standards for patient selection, optimal timing, and feeding protocols. As a result, decisions are based on individualized clinical judgment, underscoring the need for more comprehensive and standardized indications.

Given the lack of standardized criteria for FJ placement during SL in EGCs, this study provides a retrospective analysis of our institutional experience regarding the nutritional management and clinical outcomes in a series of patients who underwent this approach, generating preliminary evidence to better understand which patients are more likely to benefit from FJ placement. This case series is particularly notable for its nutritional focus, offering a different perspective on FJ usage than most similar case series in the literature, which tend to focus on surgical aspects.

## 2. Materials and Methods

We retrospectively reviewed medical records of adult patients with EGCs who underwent FJ placement during SL at the Fondazione IRCCS Policlinico S. Matteo from January 2022 to December 2023. All patients analyzed in this case series were included in the GAND-Gastric Network Database, which use was approved by the Ethical Committee of the Fondazione IRCCS Policlinico San Matteo (Pavia) on 6 December 2018 (protocol n. 20180010693). All patients provided written informed consent prior to participation.

### 2.1. Inclusion and Exclusion Criteria

Patients with a confirmed diagnosis of cancer of the esophagus, gastro-esophageal junction (GEJ), or stomach, who underwent FJ placement during SL between 1 January 2022, and 31 December 2023, and who had at least one nutritional assessment were included.

The FJ was placed at the surgeon’s discretion, based on an individual clinical assessment of surgical factors (e.g., risk of anastomotic dehiscence) and nutritional factors (e.g., pre-existing dysphagia or impaired nutritional status), in the absence of predefined criteria or shared cutoff values.

Patients diagnosed with metastatic disease before the SL or who did not meet the inclusion criteria were excluded.

### 2.2. Nutritional and Clinical Data

Based on the available nutritional data, we identified three timepoints: T0 (first nutritional assessment after diagnosis); T1 (nutritional assessment after FJ placement); T2 (nutritional evaluation at disease restaging).

For these timepoints we collected data from electronic medical reports and from nutritional assessments performed by Registered Dietitians (RD) of the Clinical Nutrition Unit during inpatient consultations or during outpatient nutritional visits. In case nutritional assessments were not available, we retrieved nutritional data from surgical and oncological visit reports.

Although it was not possible to evaluate body composition, we were able to retrieve data regarding: age, sex, weight, height, BMI, involuntary weight lost in the last 6 months, BMI-adjusted weight loss grading system (WLGS) [50], 2002 Nutritional Risk Screening (NRS-2002 score) [51], Malnutrition Universal Screening Tool (MUST) score [52], basal energy expenditure (BEE) (calculated through Harris–Benedict prediction equation), total daily energy expenditure (TDEE) (calculated multiplying BEE for patients’ specific activity and stress factors, according to ESPEN guidelines on nutrition in cancer patients [53]), intake of oral nutritional supplements (ONS), presence of dysphagia to solids and/or liquids.

Regarding data on FJ and enteral nutrition, we were able to identify the date of positioning of the device during the exploratory laparoscopy procedure, the date of start of home enteral nutrition, the amount of calories delivered with enteral nutrition, the percentage of coverage of the TDEE, and FJ-complications (infection, displacement, obstruction, leakage, other tube damage, resulting in or not resulting in hospital admission and/or death of the patient) or feeding intolerance with or without withdrawal of enteral nutrition.

We also recorded data on Charlson Comorbidity Index (CCI) [54], chemotherapy regimens, related toxicities and complications, as well as postoperative outcomes for patients who underwent esophagectomy or gastrectomy.

Follow up data were gathered, with the last follow-up date set for April 2024.

Data are presented as median with interquartile ranges (IQR) or as numbers with corresponding percentages (%).

## 3. Results

We identified a total of 35 patients who underwent SL from 1 January 2022 to 31 December 2023. Among these, 14 patients (40%) met all the inclusion criteria and are presented in this case series. The remaining 21 patients were excluded for the following reasons: 8 had metastatic disease and/or underwent laparoscopy for complications (e.g., bleeding); 6 underwent laparoscopy but did not place the FJ; 3 patients did not have any oncological disease; 2 patients had other kind of neoplasms; and 2 patients had no available nutritional data.

A retrospective review of the clinical discharge letters of the six patients who underwent SL without FJ placement, showed that the majority of these patients, including those with intraoperative findings of peritoneal carcinomatosis, were managed with ONS or oral immunonutrition protocols. Regarding the remaining cases, one patient presented with severe comorbidities (coagulopathy), while another was a candidate for experimental immunotherapy at an external oncology institution.

Table 1 details the baseline demographic, clinical, and nutritional characteristics of the study population at the time of diagnosis.

### 3.1. Patients Characteristics: Demographics, Cancer Diagnosis, Comorbidities

All patients were Caucasian, 12 were males (85.7%), 2 (14.3%) females. Median age at the time of the first nutritional assessment (T0) was 66 years (IQR: 56.3–69.5).

Diagnosis of EGCs were distributed as following: eight cases of GEJ cancer (57.1%), four cases of GC (28.6%), and two cases of EC (14.3%).

Eight patients (57.1%) had no comorbidities, five patients (35.7%) had one comorbidity (14.3%), including history of myocardial infarction, chronic hepatitis, chronic obstructive pulmonary disease, and uncomplicated diabetes mellitus. Only one patient presented two comorbidities which were peripheral vascular disease and history of stroke or transient ischemic attack. Median age-adjusted CCI score was 3 (IQR: 1.3–3).

### 3.2. Cancer Treatments

After SL, for 13 patients (92.8%) systemic oncologic treatments were planned. In particular, three patients (21.4%) had an indication to undergo first-line chemotherapy with oxaliplatin combined with 5-fluorouracil and folinic acid (FOLFOX-6) scheme due to evidence of metastases at SL or at CT images revision after laparoscopic exploration. One patient was prescribed a concomitant chemoradiotherapy for esophageal squamous carcinoma, although this patient never started this treatment because of early death due to septic shock unrelated to the FJ. Nine patients (64.3%) were prescribed a perioperative chemotherapy with FLOT. Seven out of nine (77.8%) completed the planned four cycles of neo-adjuvant therapy; one patient completed only one cycle of FLOT scheme and died after experiencing enteritis and septic shock, while one patient was lost to follow-up. Finally, one patient did not receive any indication for pre-operatory systemic treatments and underwent to upfront surgery due to comorbidities.

### 3.3. Nutritional Assessment

#### 3.3.1. T0—First Nutritional Assessment After Diagnosis

Median BMI at diagnosis was 23.7 kg/m^2^ (IQR: 21.6–26.3). According to BMI, eight (57.1%) patients had normal weight, four (28.6%) were overweight, one was obese (7.1%), and one was underweight (7.1%).

All patients in this series experienced some degree of involuntary weight loss. In fact, the median weight loss in the previous 6 months was 13.4% (IQR: 8.7–16.8).

To assess the severity of weight loss adjusted for BMI, we utilized the weight loss grading system (WLGS). The median WLGS score in the series was 3 (IQR: 3–4). Notably, 78.6% (n.11) of the patients scored 3 or 4 on the grading scale, which indicates a higher prognostic risk.

Both nutritional screening tools retrospectively applied to nutritional data reported at the time of diagnosis revealed in more than half of patients of this case series a high risk of malnutrition: with median NRS-2002 = 3 (IQR:2–4) and MUST = 2 (IQR:1–2).

Dysphagia to solids was present in seven patients (50%), while dysphagia to both solids and liquids was present in three patients (21.4%). Only four (28.6%) did not manifest dysphagia.

Only four (29%) patients were using ONS at T0, providing a median of 21.5% of energy requirements (IQR: 16.4–25.8). All ONS were ready-to-drink liquid products, except for one patient using a ready-to-eat creamy ONS.

Nine patients (64%) were evaluated by a RD 9 days (IQR: 6–13) before FJ placement. In two patients (14.3%), nutritional evaluation was performed on the day of surgical procedure, while in three cases (21.4%) it was delivered a few days after.

The median BMI at time of the positioning of FJ was 23.7 kg/m^2^ (IQR: 21.3–24.6).

#### 3.3.2. T1—Nutritional Assessment After FJ Placement

**Figure 1:**
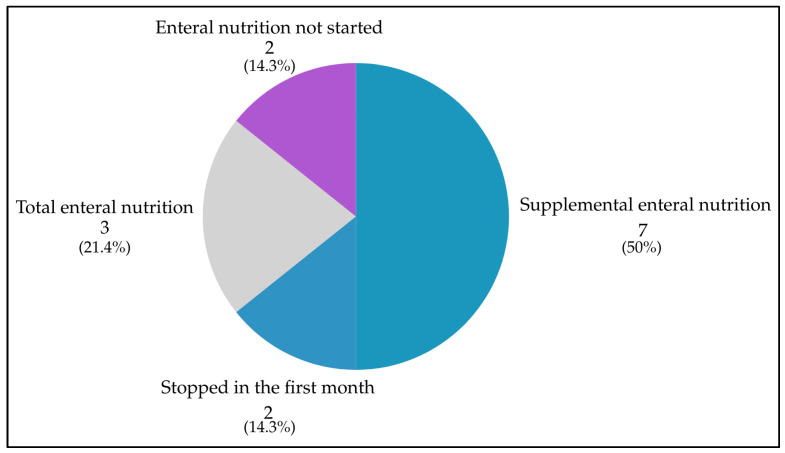
Home enteral nutrition use after FJ placement in the case series (n = 14). Numbers indicate the number of patients in each category, with corresponding percentages.

Home enteral nutrition (HEN) was started soon after FJ placement in 12 (85.7%) cases: 9 (64.3%) as supplemental HEN providing a median of 45.5% of energy needs (IQR: 32.6–68.2); 3 (21.4%) as total enteral nutrition. Due to adequate oral intake, in two (14.3%) cases, HEN was not started, and in two (14.3%) cases on supplemental HEN it was stopped in the first month, after 2 days in one case, and after 18 days in another case. The distribution of HEN initiation and discontinuation is detailed in Figure 1.

More than half of the patients (51.1%, n.8) maintained their weight at T1 compared to T0; four patients (28.6%) lost a median of 5.4% of their body weight (IQR: 3.2–8.3). Only one patient gained weight.

#### 3.3.3. T2—Nutritional Evaluation at Restaging

For this timepoint, data are missing for three patients: case 3 and 14 died, while case 6 was lost to follow-up.

The median BMI at time of the end of chemotherapy or at surgery was 23.8 kg/m^2^ (IQR: 20.4–24.7). Compared to T0, six patients (42.8%) lost a median of 8.4% of their body weight (IQR: 6.7–9.8) at T2, two (14.3%) patients achieved weight stability, while a weight gain of 4.5% (IQR: 3.8–6.7) was observed in three (21.4%).

At T2, six patients (42.8%) were continuing HEN, providing a median of 69.6% of energy needs (IQR: 36.9–93.8).

### 3.4. Feeding Jejunostomy Use

The patterns of FJ utilization are analyzed in Table 2, where patients are stratified according to the duration of HEN to highlight differences between less than one month users and more than one month users.

**Figure 2:**
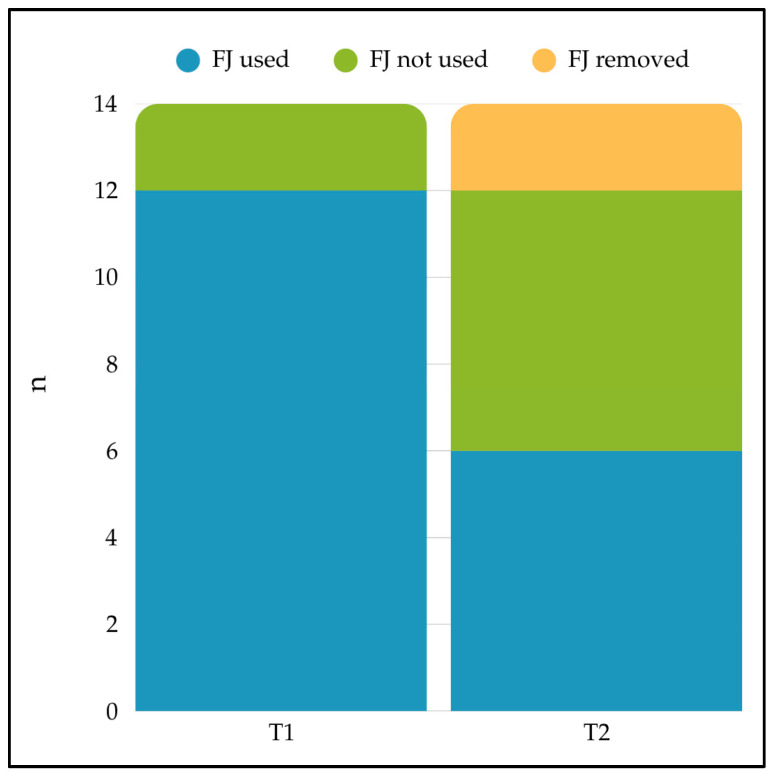
FJ use in the case series at T1 and T2.

FJ was used by 12 patients (85.7%) at T1, while its use at T2 was recorded in only half of them (6 patients, 42.8%). The evolution of FJ usage status between the two timepoints is illustrated in Figure 2. The median duration of enteral nutrition provided by FJ, for patients using it, was 97 days (IQR: 40–135).

Among FJ users at T2, compared to T1, an even distribution of weight patterns was observed: weight gain in two patients (14.3%; +3% and +8.2% of body weight), weight stability in two patients (14.3%), and weight loss in two patients (14.3%; −2.7% and −10.2% of body weight).

A temporary suspension of enteral nutrition for more than 2 weeks was prescribed in one case (7.1%) due to intestinal perforation. In one case (7.1%), enteral nutrition formula was changed because of intolerance (nausea and diarrhea).

FJ was maintained in place in all cases for a median of 126 days (IQR: 94–168). In the two patients from the series who never activated HEN, FJ was maintained in place while not using it for 91 and 94 days, respectively. Meanwhile, in the other two cases where HEN was stopped early (i.e., in the first month), FJ was retained without use for 63 and 286 days.

Among the five patients who died during the study period (35.7%), the FJ was not removed until death; of these, three (60%) were still using it, while two (40%) were not using it.

Considering the whole series, FJ was retained but not used for 11 days (IQR: 3–91). In some of the cases, FJ was maintained for precautionary purposes only.

FJ complications requiring hospitalization occurred in three patients (21%) and were represented by leakage, dislodgement, infection, and device damage.

### 3.5. Mortality

Vital status was recorded for 13 patients (92.8%); for one patient, it was not recorded due to loss at follow-up. After a median follow-up of 235 days (IQR: 92–416) from the first nutritional assessment, five patients died resulting in a mortality rate of 35.7%. Death occurred in hospice or home palliative care settings in three cases; one patient died in the intensive care unit from an unknown cause and one patient died in the oncology unit due to septic shock unrelated to FJ. A median of 97 days (IQR: 90–174) elapsed between the first nutritional assessment and death. Eight patients (61.5%) were still alive and were undergoing active anticancer treatment or follow-up visits, after a median of 407 days (IQR: 245–471) from the first nutritional assessment.

## 4. Discussion

In our case series, the placement of a FJ during SL was linked to a relatively low incidence of serious complications; however, the clinical benefit appeared uncertain for several patients due to low usage rates. Many patients had the FJ for months without utilizing it and some patients had not undergone ONS or received evaluation from a dietitian before the placement of the FJ. Notably all patients experienced some degree of involuntary weight loss before EGC diagnosis, as expected due to the location of the disease. In fact, in EGC patients weight loss commonly occurs due to various factors associated with the disease and its treatment, including dysphagia, mechanical obstruction at the tumor site, cancer-related cachexia and malnutrition, treatment side effects such as nausea, vomiting, anorexia, and altered taste perception, and psychological symptoms.

More than half of the patients in the series were at high risk of malnutrition, although not all of them had been evaluated by a dietitian prior to FJ placement. Moreover, prior to FJ placement, only four patients had tried ONS.

Consistent with the GLIM framework introduced above, our cohort exhibited a high prevalence of malnutrition based on phenotypic (weight loss, BMI) and etiologic factors. However, due to the retrospective design, data on muscle mass reduction (sarcopenia) were unavailable. Since nearly all patients (12 out of 14) met the GLIM criteria for malnutrition, a comparative analysis between malnourished and non-malnourished subgroups was not feasible.

Nutritional screening is a fundamental aspect of the management of cancer patients, as emphasized by the guidelines provided by the European Society for Clinical Nutrition and Metabolism (ESPEN) on nutrition and cancer [53]. Early identification of malnutrition together with appropriate nutritional interventions is crucial for improving clinical outcomes in these patients. According to ESPEN guidelines, the additional use of ONS is advised when an enriched diet, as a result of nutritional counseling, is not effective in reaching nutritional goals. Consequently, well-tolerated ONS can improve the nutritional status of cancer patients. Interestingly, in our case series, ONS utilization potentially could have questioned the necessity of FJ placement if these had been timely tested.

At the time of FJ placement, more than half of the patients maintained their weight compared to baseline. This is probably due to the short interval of time typically elapsed between nutritional assessment at diagnosis and nutritional assessment after FJ placement. However, four patients (28.6%) continued to lose weight.

Home enteral nutrition (HEN) was started soon after FJ placement in the majority of patients, mainly as supplemental HEN providing on average 50% of energy requirements and in a minority as total enteral nutrition. However, we observed that in two cases (14.3%) HEN was not started, and in another two cases (14.3%) it was stopped early. These cases probably represent improper placements that expose the patient to risks and yield no benefits. Risks include procedural risks (infections, bleeding), discomfort and distress for the patient, as well as inappropriate use of hospital resources. These could be reduced by providing routine patient and caregiver education when HEN is initiated and by ensuring proper monitoring as suggested by international guidelines [55].

In some cases, FJ was never used or used for only a few days; furthermore, many patients had an FJ for months without using it before its removal, exposing patients to all risk mentioned above and besides not benefiting from it. Finally, FJ-related complications requiring hospitalization occurred in three (21.4%) patients.

At the end of neoadjuvant chemotherapy, weight loss was still observed in six (42.8%) patients compared to baseline. This percentage is slightly higher than that reported in other studies, which found weight loss during HEN in 26% of patients [30]. In our case series, four out of six (66.7%) patients who lost weight had undergone HEN, while all five patients (100%) with stable/increased weight had undergone HEN.

Unfortunately, despite the presence of a FJ, nutritional evaluations were not regularly performed for the patients retrospectively included in this case series. This may have contributed to suboptimal management of nutritional support in these patients and is consistent with data on nutritional screening in Italy, which report that it is performed in only around 30% of cancer patients [27]. Regular nutritional reassessments should be provided throughout the anticancer treatment path (before, during and after oncological treatment) at least in patients with cancers known to be associated with a high prevalence of malnutrition, including EGCs. Oral food intake should also be frequently monitored, and whenever possible, body composition should be assessed. In a multidisciplinary approach, these data are crucial for defining the optimal nutritional support and specifically when FJ placement might truly be beneficial.

This study has several limitations. First, the retrospective single-center design and the small, heterogeneous sample size limit the generalizability of our findings. Second, a critical limitation is the absence of a control group. Although six patients underwent staging laparoscopy without FJ placement during the same period, they were excluded to avoid statistically unreliable comparisons given the limited sample size; consequently, no comparative inferences regarding nutritional outcomes could be drawn. Third, regarding data completeness, body composition analysis, specific laboratory markers, and functional capacity assessments were not available. Furthermore, while most nutritional data were derived from standardized assessments by Registered Dietitians, the reliance on surgical or oncological reports for some missing data points introduces a risk of heterogeneity and information bias. Fourth, several patients were lost to follow-up, and the relatively short follow-up duration prevents the assessment of long-term survival outcomes.

However, our findings support the need for standardized patient selection for FJ placement to help clinicians to decide whether or not to place a FJ when the risks and benefits do not clearly favor one choice over the other. Additionally, routine preoperative nutritional assessments for SL in EGCs should be implemented, in order to timely identify patients at high risk of malnutrition and promptly start the more appropriate nutritional support. A recent publication described an algorithm proposal to identify patients who would most likely benefit from FJ placement at different timepoints throughout the EGC care journey [56]. In particular, for FJ placement during SL, patients were stratified, based on the Nutritional Risk Screening-2002 (NRS-2002) score, into high-risk (NRS-2002 ≥ 3) and low-risk (NRS-2002 ≤ 2) groups. According to this proposal, high-risk patients should receive oral nutritional supplementation unless contraindicated (e.g., severe dysphagia); while FJ placement should be recommended when supplementation is inadequate or when overt malnutrition is present (BMI < 20, weight loss > 10% over the previous three months, or NRS-2002 ≥ 5); conversely, low-risk patients should undergo regular nutritional surveillance; and finally, FJ placement should be considered for patients with declining oral intake and/or decreasing muscle mass if oral nutritional supplementation is insufficient.

## 5. Conclusions

EGCs can profoundly affect the nutritional status of patients at any stage of the disease. It is crucial for these patients to be promptly referred to comprehensive nutritional programs within a multidisciplinary setting to early detect and treat malnutrition with appropriate nutritional support.

In this retrospective analysis of our institutional experience, we described the patterns of utilization of a FJ, placed during SL, in a series of 14 cases, contributing in highlighting the need for standardized criteria for patient selection and implementation of regular nutritional assessments in clinical practice.

These preliminary observations serve as a rationale for future larger observational prospective study to compare patients who did not undergo FJ placement and patients who did, and analyzing the impact of FJ on surgical outcomes, as well as clinical and nutritional outcomes.

Finally, if standardized procedures for nutritional support through FJ placement for EGCs patients are established, it would be useful to set up a prospective interventional study to analyze their impact on oncological and surgical outcomes.

## Figures and Tables

**Table 1:** Patients’ characteristics at first nutritional assessment (T0).

Case (Gender, Age)	Tumor Site	Dysphagia	Comorbidities	n. of Days Between T0 and Death	Pre-Illness BMI	BMI at T0	Weight Loss (%)	NRS-2002 Score	MUST Score
n.1 (M, 70)	Stomach	No	None	-	26.2	22.3	14.9	4	1
n.2 (M, 66)	GEJ	Dysphagia to solids and liquids	COPD	-	30.5	23.7	22.1	4	4
n.3 (M, 61)	GEJ	Dysphagia to solids	Peripheral vascular disease, history of stroke or TIA	90	28.4	26.5	6.5	2	1
n.4 (M, 80)	GEJ	Dysphagia to solids	None	-	27.7	24.6	11.3	5	2
n.5 (M, 68)	Stomach	No	Myocardial infarction	-	29.6	28.4	4.2	1	0
n.6 (M, 66)	GEJ	Dysphagia to solids	myocardial infarction	-	33.2	27.7	16.5	3	2
n.7 (M, 56)	Stomach	Dysphagia to solids	None	-	26.6	23.4	12	2	2
n.8 (M, 70)	Esophagus	Dysphagia to solids	None	-	24.7	23.7	4.3	2	0
n.9 (M, 47)	GEJ	Dysphagia to solids	Chronic hepatitis	97	27.8	25.5	8.2	2	1
n.10 (M, 57)	EGJ	No	None	-	33.8	30.4	10	2	1
n.11 (F, 48)	Esophagus	Dysphagia to solids and liquids	None	60	27.7	21.4	22.9	4	4
n.12 (M, 67)	GEJ	Dysphagia to solids	Uncomplicated diabetes mellitus	-	25.1	21.3	15.1	3	2
n.13 (F, 74)	Stomach	Dysphagia to solids and liquids	None	174	22.3	17.6	21.1	5	6
n.14 (M, 46)	GEJ	No	None	297	25.4	21.1	16.9	3	2

EGJ: gastro-esophageal junction; COPD: Chronic obstructive pulmonary disease; TIA: transient ischemic attack.

**Table 2:** Patterns of Feeding Jejunostomy (FJ) use in the case series stratified by duration of Home Enteral Nutrition.

Case	Duration of Home Enteral Nutrition -HEN- (days)	FJ’s Use at T1	FJ’s Use at T2	FJ-Related Complications Requiring Hospitalization	Retention of FJ from Placement to Removal (Days)
HEN users for less than one month or non-users					
n.5	no (never activated HEN)	no	no (never activated HEN)	none	91
n.10	no (never activated HEN)	no	no (never activated HEN)	none	94
n.14	2	yes	death	none	288
n.3	18	yes	death	displacement	81
HEN users for more than one month					
n.8	36	yes	no	none	167
n.11	43	yes	yes	none	54
n.9	93	yes	yes	none	97
n.7	97	yes	yes	none	98
n.12	121	yes	yes	leakage	126
n.1	129	yes	yes	none	208
n.2	141	yes	no	none	143
n.13	167	yes	yes	none	168
n.4	217	yes	no	infection and damage	220
HEN users with incomplete data					
n.6	yes	missing	none	missing	missing

## Data Availability

The raw data supporting the conclusions of this article will be made available by the authors on request.

## Abbreviations

The following abbreviations are used in this manuscript:

EGCs	Esophagogastric cancers
FJ	Feeding Jejunostomy
SL	Staging Laparoscopy
T0	First nutritional assessment after diagnosis
T1	Nutritional assessment after FJ placement
T2	Nutritional evaluation at restaging
WLGS	Weight Loss Grading system
NRS-2002	2002 Nutritional Risk Screening
MUST	Malnutrition Universal Screening Tool
BEE	Basal Energy Expenditure
TDEE	Total Daily Energy Expenditure
ONS	Oral Nutritional Supplements
HEN	Home enteral nutrition
